# Assessment of nurse’s knowledge about Glasgow coma scale at a university hospital

**DOI:** 10.1590/S1679-45082016AO3618

**Published:** 2016

**Authors:** Wesley Cajaíba Santos, Cássia Regina Vancini-Campanharo, Maria Carolina Barbosa Teixeira Lopes, Meiry Fernanda Pinto Okuno, Ruth Ester Assayag Batista

**Affiliations:** 1 Universidade Federal de São Paulo, São Paulo, SP, Brazil.

**Keywords:** Nursing assessment, Knowledge, Nurses, Glasgow coma scale, Consciousness disorders

## Abstract

**Objective:**

To assess knowledge of nurses of emergency services and intensive care units about Glasgow Coma Scale.

**Methods:**

This cross-sectional analytical study included 127 nurses of critical units of an university hospital. We used structured interview with 12 questions to evaluate their knowledge about the scale. Association of Knowledge with professionals’ sociodemographic variables were verified by the Fisher-test, χ^2^ and likelihood ratio.

**Results:**

Most of participants were women mean aged 31.1 years, they had graduated more than 5 years previously, and had 1 to 3 years of work experience. In the assessment of best score possible for Glasgow scale (question 3) nurses who had graduate more than 5 years ago presented a lower percentage success rate (p=0.0476). However, in the question 7, which evaluated what interval of the scale indicated moderate severity of brain trauma injury, those with more years of experience had higher percentage of correct answers (p=0.0251). In addition, nurses from emergency service had more correct answers than nurses from intensive care unit (p=0.0143) in the same question. Nurses graduated for more than 5 years ago had a lower percentage of correct answers in question 7 (p=0.0161). Nurses with more work experience had a better score (p=0.0119) to identify how assessment of motor response should be started.

**Conclusion:**

Number of year since graduation, experience, and work at critical care units interfered in nurses’ knowledge about the scale, which indicates the need of training.

## RESUMO

**Objetivo:**

Avaliar o conhecimento de enfermeiros de unidades críticas, serviços de emergência e unidades de terapia intensiva em relação à escala de coma de Glasgow.

**Métodos:**

Estudo transversal e analítico com 127 enfermeiros de unidades críticas de um hospital universitário. Utilizou-se entrevista estruturada com 12 questões que avaliaram conhecimento sobre a escala. Associação do conhecimento com variáveis sociodemográficas dos profissionais foi verificada pelo teste de Fisher, teste χ^2^ e razão de verossimilhança.

**Resultados:**

Houve predominância de mulheres, média de idade de 31,1 anos, especialistas, mais de 5 anos de formado e experiência profissional de 1 a 3 anos. Na avaliação do melhor escore possível para pontuação na escala (questão 3), enfermeiros com tempo de formação maior que 5 anos apresentaram menor porcentual de acertos (p=0,0476). Em relação à questão 7, que avaliou qual intervalo da escala indicava gravidade moderada do trauma craniencefálico, observou-se que quanto maior o tempo de experiência, maior o porcentual de acertos (p=0,0251), sendo que enfermeiros do serviço de emergência tiveram mais acertos nessa questão em relação aos das unidades de terapia intensiva (p=0,0143). Além disso, enfermeiros formados há mais de 5 anos apresentaram menor porcentual de acertos nessa questão (p=0,0161). Quando se identificou como deve ser iniciada a avaliação da resposta motora, enfermeiros com maior tempo de trabalho na unidade apresentaram mais acertos (p=0,0119).

**Conclusão:**

Tempo de formação, experiência e trabalho na unidade interferiu no conhecimento de enfermeiros sobre a escala, evidenciando necessidade de capacitação.

## INTRODUCTION

Traumatic injuries are important cause of morbidity and mortality around the world, and the brain trauma injury (BTI) is one of the main determinants of this panorama. The BTI has complex physiopathological mechanisms.^(1-3)^ Among non-traumatic causes of consciousness change, we highlight abscesses, brain tumors, injuries from strokes, hydrocephalies and aneurism, among other, which are also called structure injuries. Among non-structural injuries most prominent are hypoxia, hydroelectrolytic changes, hyperthermia and hypothermia, hepatic encephalopathy, alcohol intoxication, illicit drugs, hypnotic sedatives and heavy metals.^(4,5)^

Glasgow coma scale (GCS), developed by Taesdale and Jennet in 1974 at The University of Glasgow, Scotland, UK, is employed worldwide to identify neurologic dysfunction and follow-up progress of level of consciousness, predict prognosis, and standardize communication among health professionals.^(6)^ This scale became an important tool to assist patient who suffered trauma, mainly BTI victims, and, posteriorly, its use extended to other neurologic conditions that can alter consciousness. Total score ranges from 3 to 15 and it is obtained by observation of spontaneous activities and use of verbal and/or painful stimulus.

The GCS is divided in three assessment parameters:

– Eye opening (score 1 to 4): spontaneous eye opening when standing next to the patient’s bed or even during procedures receives a score of 4. Eye opening by verbal stimulus using the simple commands such as “open your eyes”, sometimes-continuous verbal stimulus is needed, the score given is 3. Eye opening with painful stimulus, applied by the examiner, in regions as nail bed and by supraorbital pressure, the score given is 2. No eye opening even after application of all previous described stimuli, the score given is 1.

– Verbal response (score 1 to 5): patient is oriented in time, space and aware of the self, he/she is able to answer accordingly simple questions such as “Do you know where you are right now?”, “Are you aware of what has happened?”, the score given is 5. Patient can answer questions, but incoherently, he/she is disoriented and confused, the score given is 4. A score of 3 is given for patients who answers do not match questions. The need of painful stimulus that answers are incomprehensible songs, *e.g.*, moaning, groaning, the score given is 2. No response even after application of all previous described stimuli, the score given is 1.

– Motor response (score 1 to 6): score of 6 is given for patient who obey simple commands, such as “raise your arm or leg”, “Move your feet or hands” with adequate motor response. After a painful stimulus, patient find the origin and try to remove what is causing the pain, the score given is 5. After a painful stimulus, the patient is able to find the pain and move the limb by flexion, however, he/she is not able to remove the source of pain, the score given is 4. A score of 3 is given for the patient who motor response is by flexion movement, evidenced by decortication response, therefore, presenting arms flexed, or bent inward on the chest, hands clenched into fists, and legs extended and feet turned inward. A score 2 is given for patients that motor response is by extensor movement and decerebrate posture in which neck is extended, arms are rigid extended close to elbows, legs are extended on knees level, and feet in plantar flexion. A score 1 is given for patient who does not present no motor response even after application of all previous described stimuli.^(7)^

The use of GCS requires previous knowledge and skills. This scale applied carefully and systematically is fundamental for assessment and establishment measures of the patient in order to guarantee reliability – which is critical to follow-up the progress of such patients.^(8)^

For many years, a variety of studies were developed to evaluate the precision and reliability of GCS.^(3,9)^ Studies shown low adherence of GCS use, difficulties in its application and fails of professionals related to conscience evaluation such as lack of standards and poor knowledge about the scale, in addition, the hospital routine is a fact that lead prioritization of other organic systems, indicating that only 42.7% of nurses use this scale to assess consciousness. To assess level of consciousness is part of health professionals routine, mainly those working at critical care units, *i.e.*, emergency services (ES) and intensive care units (ICU), whom are well trained and more experienced in the use of GCS with higher levels of reliability and precision.^(7,10)^

An international study that evaluated knowledge of nurses about GCS and associated results with demographic variables, reported area of working and time of experience are associated with high or low knowledge. Nurses of neonatal intensive care unit had the highest score (12.7) whereas nurses of internal medicine had lowest score (9.7). In knowledge scale, nurses working at neurologic unit over or equal to 6 years had the highest scores (11.9), and those working for less or equal to 1 year had the lowest scores (10.0). The same study reported the need of educational interventions and design of manuals for maintenance and improvement of assessment of consciousness using the GCS.^(11)^

Given the importance of GCS as a tool for neurological assessment of patients, and the need of careful and standardized application, to evaluate health professionals’ knowledge on this scale is fundamental to guarantee uniformity, reliability and accurately in the use of GCS.

## OBJECTIVE

To assess knowledge of nurses of emergency services and intensive care units about Glasgow Coma Scale.

## METHODS

This cross-sectional and analytical study included 127 nurses of the *Hospital São Paulo*, São Paulo (SP), Brazil. The study was approved by the Ethical and Research Committee of the *Universidade Federal de São Paulo*, CAAE number 42505115.8.0000.5505. All participants signed the consent form and had their information kept confidential. We included nurses who work at ES and ICU of *Hospital São Paulo*, and excluded nurses from multidisciplinary residency programs, nurses from pediatric ES and ICU, and those on sick leave or vacation during selection of participants.

Data were collected from May to July 2015. Sociodemographic variables included were: age, sex, race, marital status and formal education. In addition, the following professional variables were verified: number of years since graduation, working area, and time working in the critical care unit. We also applied a structured closed interview in a private environment for, on average, 20 minutes, to assess nurses’ knowledge on GCS and its application to the patient. Statistical analysis was performed using the Statistical Package for the Social Sciences (SPSS), version 19 (Chicago Il, USA). Descriptive analysis of continuous categorical variables was done using the mean, standard deviation, medium, minimal and maximal for each question related to the GCS. Chi square test was used to compare formal education, number of years since graduation, experience time, unit of work, and time working in the unit using the tool. When 20% of the sample had expected value lower than five, we used the Fisher exact test or likelihood ratio. We considered significant a p≤0.05 value.

## RESULTS

Table 1 includes sociodemographic and professional data of nurses in the study. We observed predominance of women (82.7%), mean aged 31.1±5,1 years, and white (72.4%). Most of professional had post-graduation courses (89.8%), 62.2% had graduated 1 to 5 years ago, and 37.8% reported 1 to 3 years of experience as a nurse. The majority of professionals worked in the ICU (76.4%) and 44.9% worked in the unit for 1 to 3 years.

**Table 1:** Sociodemographic and professional data of nurses at emergency service and intensive care unit

Variables	n (%)
Age	
Mean (standard deviation)	31.13 (5.18)
Medium	30
Minimal-maximal	22-48
Sex	
Female	105 (82.7)
Male	22 (17.3)
Race	
Black	9 (7.1)
Asian	8 (6.3)
Pardo	18 (14.2)
Marital status	
Married	34 (26.8)
Single	78 (61.4)
Divorced	4 (3.1)
Stable union	11 (8.7)
Formal education	
Graduation	10 (7.9)
Post-graduation course	114 (89.8)
Master’s degree	3 (2.4)
Number of years since graduation	
<1	1 (0.8)
1-3	42 (33.1)
3-5	37 (29.1)
>5	47 (37)
Number of years of experience	
<1	6 (4.7)
1-3	48 (37.8)
3-5	29 (22.8)
>5	44 (34.6)
Unit of work	
Emergency unit	30 (23.6)
Numbers of years working in the unit	
<1	22 (17.3)
1-3	57 (44.9)
3-5	16 (12.6)
>5	32 (25.2)

Table 2 shows professional answers related to the GCS.

**Table 2:** Description of nurses’ answers from emergency service and intensive care units related to Glasgow coma scale

Questions	n (%)
What is the function of GCS?	
Evaluate level of consciousness – right answer	126 (99.2)
Evaluate cognitive changes	1 (0.8)
Three indicators of GCS are:	
Eye opening, pupil reaction, and best motor response	1 (0.8)
Eye opening, best verbal response, and motor deficit	1 (0.8)
Eye opening, best verbal response, and best motor response – right answer	125 (98.4)
Best score for the scale is:	
10	1 (0.8)
15 – right answer	124 (97.6)
5	1 (0.8)
8	1 (0.8)
Worse score for the scale is:	
3 − right answer	124 (97.6)
4	1 (0.8)
1	2 (1.6)
GCS score that indicates critical situation and that examiner should be alert to:	
GCS ≤7	15 (11.8)
GCS ≤8 − right answer	102 (80.3)
GCS ≤15	2 (1.6)
GCS ≤5	8 (6.3)
To obtain accurate GCS results, the following criteria should be observed, expected:	
Presence of orotracheal intubation and eyelid edema	7 (5.5)
Respiratory and hemodynamic stability	4 (3.1)
Use of sedatives and neuromuscular blockade	32 (25.2)
GCS interval that indicates moderate severity is between:	
8-3	15 (11.8)
15-13	4 (3.1)
12-9 − right answer	103 (81.1)
14-8	5 (3.9)
During the use of GCS, the most adequate response for score is:	
The first response presented by the patient	8 (6.3)
Best response presented by the patient − right answer	118 (92.9)
Last response presented by the patient	1 (0.8)
To assess eye opening, examiner should begin with:	
Verbally request the patient to open his/her eyes	19 (15)
Call the patient’s name out loud	57 (44.9)
Use painful stimuli	1 (0.8)
Stand next to the patient’s bed – right answer	50 (39.4)
To assess best verbal response, examiner should begin with:	
Marking different questions	14 (11)
Simple question about localization, time, space and self – right answer	113 (89)
To assess best motor response, examiner should begin with:	
Verbal command requesting a motor response - right answer	114 (89.8)
Use of painful stimulus	4 (3.1)
Observe muscle strength	6 (4.7)
Observe range of movement	3 (2.4)
In GCS take notes for:	
Only the total score	7 (5.5)
Describe responses obtained	1 (0.8)
Scoring each indicator	5 (3,9)
Scoring each indicator, total score, and describe when necessary – right answer	114 (89,8)

GCS: Glasgow coma scale.

When professionals were inquired about the applicability of the scale, they (99.2%) reported it applies for level of consciousness. About parameters of GCS, 98.4% of professionals mentioned eye opening, best verbal response and best motor response. When professionals were inquired what is the best score possible for the scale, 97.6% of them mentioned 15, and the least score possible, 97.6% checked the score as correct.

When questioned about GCS score that indicate critically status for the patient and must be used as an alarming sign for the professional, 80.3% reported that score was ≤8. A total of 66.1% of professionals identified urinary incontinence as the aspect that influenced GCS use. The scale interval that classified BTI, according to severity, 81.1% of participants answered as correct, indicating this interval between score 12-9 for moderate BTI. Most of participant (92.9%) answered right what would be the most adequate response to be scored, *i.e.*, the best response presented by the patient.

When nurses were inquired about when to begin eye opening parameter, 39.4% answered correctly, which is to get close to patient’s bed. When participants were inquired, how examiner would begin the parameter best verbal response, 89.0% reported that questioning about time, space, and aware of the self was the right answer. A total of 89.8% of participants answered that to begin the best motor response the best approach would be verbal command by requesting a motor response.

Most of participants (89.8%) answered correctly that GCS would be recorded using the score obtained in each parameter, and by provide the total score of the scale.

When compared variables (formal education, numbers of years since graduation, time of experience, unit of work, time workingin the unit) with answer in each unit, we observed that in question 3 that evaluated the best score possible for the score in the scale, nurses who graduated more than 5 years ago had the lowest percentage of right answers (p=0.0476).

In question 7, which evaluated what interval of GCS would indicate moderate severity of BTI, the more time of experience the higher was percentage of right answers (p=0.0251), and nurses from emergency unit had the highest percentage of right answers than nurses from intensive care unit (p=0.0143). In addition, nurses who graduate for more than 5 years ago had lowest percentage of right answers in the question (p=0.0161).

Question 11, which evaluated how the examiner should begin the assessment of best motor response, nurses with more work experience in the unit had the highest percentage of right answers (p=0.0119).

## DISCUSSION

Professionals providing care for severe patients face situations with any type of neurologic disorder that may require an easy to apply instrument that should be able to identify quickly these disorders and changes in neurologic parameters (consciousness, sensibility and motricity). For this reason, neurologic assessment of these patient is an indispensable tool in daily practice at ES and ICU.^(10)^

Most of nurses included in the study were women, mean aged 31.18 years, white and single. These results corroborate, in some extent, with findings of other study carried out in the adult intensive care unit at a teaching hospital in São Paulo countryside that reported predominance of women and mean age of 32 years. In that study, age ranged between 23 and 33 years, therefore, a young population.^(12)^

Most of participants in our study had post-graduate course and reported to be graduated between 1 and 5 years previously (62.2%). A low percentage was found in research carried out in ICU of three university hospitals in Alagoas State in which 68% of nurses reported to have a post-graduate course.^(13)^ Another study carried out in cardiac intensive care unit of Hospital dos Servidores do Estado do Rio de Janeiro (RJ), observed that 50% of nurses had graduated between 1 to 5 years previously.^(14)^

In our study, the majority of nurses (more than 80%) answered correctly questions about GCS concerning the aim of the scale, parameters, score, scale score that indicated critical situation for the patient and that examiner should be alert to, and also about the scale interval that classified BTI as moderate. They also answered correctly how to score each parameter, and how to begin the assessment of best verbal response and best motor response.

Although the majority of nurses of our study showed a good knowledge about GCS, some professionals had poor knowledge about the scale. This fact corroborates with finding of an international study in which participants showed poor knowledge in some parameters of the scale, which indicated the need of continuous training in order to guarantee standard and reliable use of GCS.^(15)^ Such scenario can be a concern, especially because neurologic assessment is prevent and diagnose early events that can trigger secondary brain injuries or worsen existing injuries. Severely ill patients require pointless perception and follow-up from nurse team. Therefore, nurses should know changes that may occur with the patient in order to act correctly because nursing care is based on constant observation and correct evaluation.^(15)^

When participants in our study were inquired about correct way to assess eye opening, only 39.4% chosen the right answer. This finding was reported by another study that compared GCS application among nurses and physician from the emergency unit. In that study, participants also had low score in this parameter of the scale.^(16)^

Nurses who graduated more than 5 years ago had lower percentage of right answers in question related with score associated to interval indicating moderate severity of GCS. This result can be associated to lack of training, reliability and precision in the use of scale. Knowledge improves with experience and continuous training.^(8)^

In question that evaluated GSC interval indicating moderate severity, more experience professionals had higher percentage of right answers, and nurses from emergency unit did better compared with professional from ICU. Nurses working in critical patient care need scientific, practical and technical knowledge in order to take quick and effective decisions given that neurologic changes are related with improvement or worsening of patient’s prognosis. A safety care for patient requires skilled nurse who can perform their role to guarantee patient recovery. Care delivery by nurses requires various knowledge and understanding related with leadership process of the team, with special emphasis in interpersonal relationship and decision making.^(17)^ Professional experience along with training is fundamental in this process to guarantee quality improvement in nursing care

Nurses included in our study who worked for more time within the unit had higher percentage of right answer in question assessing how examiner should record motor response, and the same results were seen in other studies, highlighting more experience as higher knowledge about GCS^(11,18,19)^ This result shows the importance of education in services that use GCS, because most professionals working at ES are young, but they have little working experience. Another important fact is high turnover at emergency services.^(20,21)^

Considering the results of our study, we believe that training strategies on the use of GCS should be a topic of reflection to guarantee reliability and precision expected from nurse professionals.^(8)^ Simulation-based teaching constitutes an active learning method in an environment free of risk with the possibility to build knowledge and technical skills. This method can be a strategy that would provide safety for nurses in the use of GCS in clinical practice.^(22)^

The main limitation of this study is that it was conducted in a single center, therefore, it constitutes a local experience.

## CONCLUSION

Most nurses of critical care units had good knowledge about Glasgow coma scale and answered correctly questions about it, exceptions were seen about eye opening parameters.

Those with more experience as a nurse had higher percentage of right answers in the question related to Glasgow coma scale interval, which indicate moderate severity of brain injury. Nurses working for more time in the unit had higher percentage of right answers in the question inquiring how examiner should begin assessment of best motor response.
